# Prevalence and correlates of post-traumatic stress disorder and its symptomatology in tornado-affected rural residents

**DOI:** 10.3389/fpsyt.2022.946450

**Published:** 2022-08-08

**Authors:** Tai-Min Wu, Qin Yang, Yu-Jie Huang, Bao-Liang Zhong, Juan Zhang, Lian-Zhong Liu

**Affiliations:** ^1^Institute of Education, China University of Geosciences (Wuhan), Wuhan, China; ^2^Affiliated Wuhan Mental Health Center, Tongji Medical College of Huazhong University of Science and Technology, Wuhan, China; ^3^Research Center for Psychological and Health Sciences, China University of Geosciences (Wuhan), Wuhan, China

**Keywords:** tornado disaster, post-traumatic stress disorder, structured clinical interview for DSM-V (SCID), symptoms, correlates, prevalence

## Abstract

Experiencing a serious natural disaster may place survivors at particularly high risk for post-traumatic stress disorder (PTSD); however, very limited data are available on the prevalence and clinical characteristics of PTSD among survivors of a tornado disaster. The present study examined the prevalence of PTSD and correlates and clinical symptoms of possible PTSD in survivors 1.5 months after a tornado disaster. A total of 237 survivors were recruited and administered the Structured Clinical Interview for DSM-V (SCID) to measure the prevalence of PTSD and the Essen Trauma Inventory (ETI) to measure the incidence of symptoms in each dimension. Survivors'demographic information and characteristics of exposure to the tornado were collected via self-report questionnaires. Thirty-two of the survey respondents were diagnosed with PTSD (13.6%, total =237). Correlates of PTSD in survivors were being female (OR=3.62, *P* = 0.023), living in an area severely affected by the tornado (OR = 3.94, *P* = 0.032), and having severe property damage (OR = 3.72, *P* = 0.010). Less common symptoms mainly focused on the avoidance dimension and included feeling alienated or distant from the people around oneself (21.90%), not being able to recall important parts of the event (28.10%), being emotionally numb (31.20%), and feeling like one's plans for the future and hopes will not come true (37.50%). In the sample of rural residents, nearly two-thirds of people with PTSD were not willing to seek psychological help; increasing the accessibility of mental health services and administering more active mental health services are necessary for this vulnerable population, whether or not they claim to need help.

## Introduction

At 20:39 on 14 May 2021, a 17-magnitude tornado broke out in Wuhan City, Hubei Province. The wind speed reached a maximum of 60 m/s; some houses in the village bay were damaged, a large number of trees were fractured, and some work sheds collapsed. As of May 15, 2021, the tornado had killed 8 people and injured approximately 280 people. The disaster not only resulted in physical and tangible damage to the local people affected by the disaster but also caused serious psychological challenges. Relevant studies have shown that after major traumatic disaster events, disaster victims experience symptoms such as depression (1, 2), anxiety (1), sleep disturbance (3, 4), and symptoms of post-traumatic stress disorder (PTSD) (5). Severe cases are also diagnosed as PTSD (6), which greatly affects postdisaster reconstruction and the mental health of victims. From 23 June 2021 to 27 June 2021, members of the research team went to Zhashan, Caidian District, Wuhan City, Hubei Province, to conduct the research.

## Subjects and methods

The research group went to Caidian District, Wuhan City, Hubei Province from 23 June 2021 to 27 June 2021. A total of 240 questionnaires were distributed, and 237 valid questionnaires were recovered, with an effective rate of 98.3%. This study took rural residents affected by the tornado disaster as the research sample, analyzed PTSD and its symptoms in rural residents after the disaster, and analyzed its correlating factors. The detection of the symptoms of each dimension of PTSD was explored.

### Research subjects

The Respondents Were Rural Residents of three Rural Villages in Zhashan in the Tornado-affected area of Caidian District, Wuhan City. The local government classified villages according to the degree to which they were affected by the tornado: mildly affected (village A, village B, village C, village D, village E, village F, and village G) and severely affected (village H, village I and village J). According to this classification, two mildly affected villages (village B and village E) and one severely affected village (village I) of the two disaster degrees were selected as the target group by stratified sampling. In the three randomly selected villages, the method of random sampling was adopted, and 1/6 families in each village were selected as the research sample. Random sampling was carried out in each family according to the criteria of age no <12 years old and birthday close to 15 June to ensure the reliability of the sample. One sample was taken from each family.

The Institutional Review Board of Wuhan Mental Health Center approved the study protocol, and all participants signed informed consent forms.

### Research methods

The investigators were postgraduates majoring in psychology and psychiatry. All investigators underwent unified centralized training and had extensive research experience. The researchers first introduced the purpose and specific methods of the assessment to the respondents and then conducted a questionnaire survey after obtaining the respondents' informed consent. The survey was conducted in a one-on-one, question-and-answer format, and all tests were conducted using uniform guidelines. The results of the questionnaires were stored in a unified and centralized manner to ensure the confidentiality of the survey data.

### Research instruments

The research questionnaire used the Essen Trauma Inventory (ETI) to assess PTSD symptoms (7). The affected rural villagers were diagnosed with PTSD using the Structured Clinical Interview for DSM-V (SCID) (8) (the researcher conducting the PTSD diagnosis was trained in the SCID interview by the Wuhan Mental Health Center).

#### PTSD

The SCID was used to diagnose PTSD. It is the most widely used structured diagnostic tool for assessing mental illness. It can be used to assess mood disorders, psychotic disorders, substance abuse disorders, anxiety disorders, obsessive-compulsive disorders and related disorders, eating disorders, somatic symptom disorders, some sleep disorders (such as insomnia and somnolence disorders), externalizing behavior disorders (i.e., intermittent explosive disorder, gambling disorder and adult attention deficit hyperactivity disorder) and trauma and stress-related disorders.

#### PTSD symptoms

The validated Chinese version of the ETI was used to assess three symptom clusters of PTSD. The ETI was developed based on the DSM-IV-TR diagnostic criteria, and it has five parts with a total of 58 items. Part 1 contains a list of 14 potentially traumatic events and an open question asking about exposure to other traumatic events. Part 2 has 10 questions regarding the worst event's objective and subjective threat to life (criteria A1 and A2). Part 3 consists of 23 questions covering three symptom clusters of PTSD (intrusion [criteria B], avoidance [criteria C], and hyperarousal [criteria D]) and one additional symptom cluster for ASD (dissociation). Part 4 assesses the severity of psychological distress caused by the event [criteria F] and the duration of PTSD symptoms [criteria E]. Part 5 evaluates the severity of functional impairment caused by the event in terms of life satisfaction, school/work/job performance, household chores and duties, hobbies and leisure activities, relationships with friends, family relationships, and sexual life [criteria F].

#### Demographics

Demographic variables included gender, age, education, marital situation, living arrangements, personal income, smoking and alcohol consumption.

#### Exposure to the tornado disaster

Classification of the disaster degree and three questions were used to assess the severity of exposure to the tornado: “Did the disaster cause serious property damage?”, “Were you physically harmed by the disaster?”, and “Did you witness any injuries or deaths in the disaster?”.

#### Willingness to ask for psychological assistance

*Two* questions were used to assess the willingness to ask for psychological assistance: “Do you have any psychological troubles?”, “If so, do you need psychological assistance? (e.g., counseling, psychotherapy, and medication).”

### Statistical analysis

The questionnaire data were analyzed using SPSS 21.0 statistical software. Before data analysis, the normality of the data was tested by combining histograms and P-P plots of the data distribution, and the results showed that the data generally followed a normal distribution. Detection rates for PTSD were calculated, and rates between subgroups were compared using chi-square tests based on demographic and tornado disaster exposure characteristics. Significant factors from the chi-square test were included in binary logistic regression to determine the factors associated with PTSD. Associations between correlates and PTSD were quantified using OR values and 95% confidence intervals. PTSD symptoms in affected villagers were described using frequencies and percentages. A two-sided *P* < 0.05 was considered statistically significant.

## Results

### General information of all respondents

Among the 237 affected rural villagers surveyed, information on their demographics (eight items) and tornado exposure characteristics (four items) was collected with a total of 12 items. The general information is shown in Table 1. Of all survey respondents, 32 were diagnosed with PTSD based on the SCID, accounting for 13.6% of the total survey population. Of the 95 most severely affected samples, 27 (28.4%) were diagnosed with PTSD. It was found that people with PTSD were more likely to be willing to seek psychological help (psychological counseling, psychotherapy, psychopharmacological treatment) than those without PTSD (χ^2=^ 35.075, *P* < 0.001), but nearly 2/3 of people with PTSD (68.75%) still did not seek help (Table 2).

**Table 1:** Demographic information and characteristics of exposure to the tornado of rural residents who survived the tornado disaster.

**Factors**		***n***	**Ratio(%)**
**Demographic**			
Gender	Male	129	54.70%
	Female	107	45.30%
Age	28-40	14	5.90%
	41-60	97	41.10%
	61-83	125	53.00%
Education	Illiterate	28	11.90%
	Primary school	68	28.80%
	Junior middle school	95	40.30%
	High school	40	16.90%
	Undergraduate and above	5	2.10%
Marital status	Married	205	87.20%
	Unmarried	8	3.00%
	Divorced	7	3.00%
	Widowed	16	6.80%
Living arrangements	Living alone	27	11.40%
	Group quarters	2	0.80%
	Living with relatives	205	86.50%
	Other	2	0.80%
Personal monthly income	Low	156	66.40%
	Middle	76	31.90%
	High	4	1.70%
Smoking	Never or hardly smoke	162	68.70%
	Quit smoking	9	3.80%
	Smoking	65	27.50%
Frequency of alcohol consumption	Never	184	78.00%
	Occasionally	38	16.10%
	Often	8	3.40%
	Almost every day	6	2.50%
**Exposure to the tornado disaster**			
Disaster degree	Living in a mildly affected area	141	59.70%
	Living in a severely affected area	95	40.30%
Property damage	Slight property damage	161	68.20%
	Severe property damage	75	31.80%
Physical injury	Yes	11	4.70%
	No	225	95.30%
Witness any injuries or deaths	No	193	81.80%
	Yes	43	18.20%

*n* = 23.

**Table 2:** Results of the willingness to ask for psychological assistance.

		**Willingness to ask for** **psychological assistance**		
		**No**	**Yes**	**Total**
**diagnosed with PTSD**	Negative	198	6	204
	Positive	22	10	32
Total		220	16	236

### Analysis of the factors correlated with PTSD

The results of the chi-square test showed that those who were female, had severe property damage, were physically injured, were severely affected by the disaster, and witnessed injuries or deaths were more likely to have a diagnosis of PTSD. (*P* < 0.05) (Table 3).

**Table 3:** Results of the chi-square test on collected factors in rural residents who survived the tornado disaster.

**Factors**		***n***	**Number of persons diagnosed with PTSD (%)**	***X^2^***	***P***
**Demographic**					
Gender	Male	129	9(6.98%)	10.52	0.001
	Female	107	23(21.50%)		
**Exposure to the tornado disaster**					
Property damage	Slight property damage	161	11(6.83%)	19.56	<0.001
	Severe property damage	75	21(28.00%)		
Physical injury	Yes	11	6(54.55%)	16.54	<0.001
	No	225	26(11.56%)		
Disaster degree	Living in a mildly affected area	141	5(3.55%)	29.96	<0.001
	Living in a severely affected area	95	27(28.42%)		
Witness any injuries or deaths	No	193	22(11.40%)	4.22	0.040
	Yes	43	10(23.26%)		

The inclusion of the factors associated with the diagnosis of PTSD from the chi-square test results in a binary logistic regression showed that being female (OR = 3.62, *P* = 0.023), living in area severely affected by the tornado (OR = 3.94, *P* = 0.032) and having severe property damage (OR = 3.72, *P* = 0.010) were risk factors for the diagnosis of PTSD (Table 4).

**Table 4:** Results of binary logistic regression on factors associated with PTSD in rural residents who survived the tornado disaster.

**Factors**		**OR(95%)**	***P***
**Demographic**			
Gender	Male		
	Female	2.82(1.13,7.01)	0.026
**Exposure to the tornado disaster**			
Disaster degree	Living in a mildly affected area		
	Living in a severely affected area	4.51(1.46,13.92)	0.009
Property damage	Slight property damage		
	Severe property damage	3.72(1.38,10.05)	0.010

### Detection of PTSD symptoms by symptom cluster

Table 5 shows the detection of symptoms for each dimension of PTSD among the respondents. The most common symptoms in the intrusion, avoidance and hyperarousal dimensions were feeling emotionally upset when one was reminded of the event (75.00%), trying to avoid situations that reminded one of the event (56.30%), and being overly alert (75.10%), respectively. Less common symptoms mainly focused on the avoidance dimension and included feeling alienated or distant from the people around oneself (21.90%), not being able to recall important parts of the event (28.10%), being emotionally numb (31.20%), suddenly feeling like one is living through the event again (31.30%), and feeling like one's plans for the future and hopes will not come true (37.50%). See Table 5.

**Table 5:** PTSD symptoms of rural residents who survived the tornado disaster.

**Symptom**	***n*(often or very of 10)**	**%**
**Intrusion**		
Did the event cause upset thoughts or images that came to your mind although you didn't want them to?	18	56.20%
Did you have nightmares about the event?	13	40.70%
Has it ever happened to you that you suddenly felt like you were living through the event again?	10	31.30%
Did you feel emotionally upset when you were reminded of the event (feeling helpless, angry, sad, guilty)?	24	75.00%
Have you ever had physical reactions when you were reminded of the event (e.g., uneasiness, chills, or fast heartbeat)?	16	50.10%
**Avoidance**		
Have you tried not to think about the event, not to talk about it, or to suppress feelings about it?	15	46.90%
Did you try to avoid situations that remind you of the event (e.g., activities, people, or places)?	18	56.30%
Were you unable to remember an important part of the event?	9	28.10%
Did you lose interest in activities that had been important to you before the event took place (e.g., hobbies, sports)?		
Did you feel alienated or isolated from people in your environment?	7	21.90%
Did you feel emotionally numb (e.g., being unable to cry or unable to have positive feelings)?	10	31.20%
Did you feel like your plans for the future and hopes would not come true (e.g., to start a family, less luck in life or in business than the others)?	12	37.50%
**Hyperarousal**		
Did you have trouble falling or staying asleep?	21	65.60%
Did you have fits of rage or were you often nervous?	14	43.70%
Did you have trouble concentrating (e.g., forgetting what you just wanted to do or forgetting what you just read or what you saw on television)?	13	40.60%
Were you overly alert (e.g., checking to see who is around you, having a phone close-by to call for help if necessary)?	24	75.10%
Were you easily startled or highly nervous (e.g., by loud noises)?	19	59.40%

## Discussion

This study was a field study in the affected rural villages of Caidian District conducted 1.5 months after the tornado in Caidian District, Wuhan City. The prevalences of PTSD were found to be 13.6% overall and 28.4% in the most severely affected rural villages. Risk factors for PTSD diagnosis were being female, living in a severely affected area, and having severe property damage. Compared to the typical PTSD symptoms according to the DSM-V-TR criteria, the avoidance dimension was less frequent than the other two dimensions, particularly feeling alienated or distant from the people around oneself (21.90%), not being able to recall important parts of the event (28.10%), and being emotionally numb (31.20%).

In the general Chinese population, the 1-month prevalence of PTSD was 0.195% (9), which was significantly lower than the 1-month prevalence of PTSD in the affected villagers in this study (13.60%). Previous studies on the detection rate of PTSD after disaster events have also shown varying results, with the prevalence of PTSD after earthquakes, rockfalls, mudslides and car accidents ranging from 18.80 to 43.00% (10–13). The detection rate of PTSD among the affected villagers in this study was slightly higher than the rates after tornado disasters in previous studies (14, 15). The reasons for this phenomenon may be related to the status and older age of rural residents. First, rural elderly people have more severe mental health problems overall than urban elderly people (16). Second, the affected villagers were generally older (60.60 ± 11.70). The relationship between PTSD and age in previous studies is complex and may be related to the social, economic and cultural aspects of the affected areas (17), which may indicate that PTSD shows a positive correlation with age in rural areas of China. This also suggests that we need to consider the corresponding characteristics of the population when carrying out postdisaster psychological reconstruction.

Among the demographic factors, women were at significantly higher risk for PTSD than men, which is consistent with previous research findings (18). Evidence suggests that direct trauma from disasters is an important determinant of PTSD development (18, 19). The results of this study also suggest that the degree of the disaster and property damage from the tornado disasters are significantly associated with PTSD diagnosis. In particular, the prevalence of PTSD was significantly higher in the sample from severely affected areas with severe property damage than in the sample from mildly affected areas with mild property damage. In addition, the study also investigated the willingness of the sample to seek psychological help. The results show a low overall willingness to seek help among affected people, reflecting the fact that crisis interventions may not be well received by some rural residents in the aftermath of a tornado disaster.The results also showed that individuals diagnosed with PTSD were more likely to seek psychological assistance, which could include counseling, psychotherapy, and medication. This suggests that PTSD patients suffer from the pain generated by the illness, which to some extent motivates their willingness to seek psychological assistance. In postdisaster psychological reconstruction, it is important to focus on and properly deal with victims who need help, as they are more likely to be potential PTSD sufferers. Although people with PTSD are more likely to be willing to seek psychological help, nearly two-thirds of people with PTSD are not. This may be due to a sense of stigma or a lack of knowledge about psychological assistance. Therefore, in addition to increasing the accessibility of mental health services and administering more active mental health services after a disaster, publicity and dissemination of mental health knowledge should be enhanced in rural areas before a disaster occurs to raise rural residents' awareness of mental health and crisis intervention with a view to reducing their sense of stigma about psychological problems.

In general, symptoms of PTSD vary from person to person, but intrusion, avoidance, and hyperirritability are typical symptoms (19). In the present study, most PTSD symptoms were present among the affected villagers according to the DSM-V-TR criteria, but symptoms in the avoidance dimension were less common than other symptoms. This may be because the damage to their houses caused by the disaster, as well as physical injuries, were real situations that they needed to face continuously and could not escape. In addition, interpersonal interaction was higher in rural areas than in urban areas, and the process of interacting with each other made it easier for individuals to deal with the disaster that had occurred.

This study has some limitations. First, this study adopted a cross-sectional study design, but the development of PTSD and its influencing factors are complex processes that change over time. Therefore, a follow-up longitudinal study can be carried out. Second, the sample size of this study is small, and more samples can be included later to increase the reliability of the research results. Third, SCID can not only diagnose PTSD, but also other mental disorders. For instance, MDD is highly prevalent among individuals that were affected by a disaster. This research only discuss the PTSD situation. Future studies could include other parts of the SCID for the diagnosis of other mental disorders. Lastly, The investigators found that social support and post-disaster negative life events had a significant impact on the affected people's understanding of the disaster, so the Social Support and Post-Disaster Negative Life Events could be surveyed to explore the impact of social support and post-disaster negative life events on changes in the mental health of the affected people.

## Data availability statement

The original contributions presented in the study are included in the article/Supplementary material, further inquiries can be directed to the corresponding author/s.

## Ethics statement

The studies involving human participants were reviewed and approved by the Institutional Review Board of Wuhan Mental Health Center. The patients/participants provided their written informed consent to participate in this study.

## Author contributions

T-MW completed the manuscript draft. All authors contributed to the article and approved the submitted version.

## Conflict of interest

The authors declare that the research was conducted in the absence of any commercial or financial relationships that could be construed as a potential conflict of interest.

## Publisher's note

All claims expressed in this article are solely those of the authors and do not necessarily represent those of their affiliated organizations, or those of the publisher, the editors and the reviewers. Any product that may be evaluated in this article, or claim that may be made by its manufacturer, is not guaranteed or endorsed by the publisher.
