# Supplementary material for: Prevalence and correlates of post-traumatic stress disorder and its symptomatology in tornado-affected rural residents
Source: Front Psychiatry. 2022 Aug 8;13:946450. doi: 10.3389/fpsyt.2022.946450 (PMC9394182; doi:10.3389/fpsyt.2022.946450)
Supplement: Supplementary file 1 [file Data_Sheet_1.ZIP › Date Sheet 1/table 1.docx]

**Table 1.** Demographic information and characteristics of exposure to the tornado of rural residents who survived the tornado disaster.

| **Factors** |  | ***n*** | **Ratio（%）** |
| --- | --- | --- | --- |
| **Demographic** |  |  |  |
| Gender | Male | 129 | 54.70% |
|  | Female | 107 | 45.30% |
| Age | 28-40 | 14 | 5.90% |
|  | 41-60 | 97 | 41.10% |
|  | 61-83 | 125 | 53.00% |
| Education | Illiterate | 28 | 11.90% |
|  | Primary school | 68 | 28.80% |
|  | Junior middle school | 95 | 40.30% |
|  | High school | 40 | 16.90% |
|  | Undergraduate and above | 5 | 2.10% |
| Marital status | Married | 205 | 87.20% |
|  | Unmarried | 8 | 3.00% |
|  | Divorced | 7 | 3.00% |
|  | Widowed | 16 | 6.80% |
| Living arrangements | Living alone | 27 | 11.40% |
|  | Group quarters | 2 | 0.80% |
|  | Living with relatives | 205 | 86.50% |
|  | Other | 2 | 0.80% |
| Personal monthly income | Low | 156 | 66.40% |
|  | Middle | 76 | 31.90% |
|  | High | 4 | 1.70% |
| Smoking | Never or hardly smoke | 162 | 68.70% |
|  | Quit smoking | 9 | 3.80% |
|  | Smoking | 65 | 27.50% |
| Frequency of alcohol consumption | Never | 184 | 78.00% |
|  | Occasionally | 38 | 16.10% |
|  | Often | 8 | 3.40% |
|  | Almost every day | 6 | 2.50% |
| **Exposure to the tornado disaster** |  |  |  |
| Disaster degree | Living in a mildly affected area | 141 | 59.70% |
|  | Living in a severely affected area | 95 | 40.30% |
| Property damage | Slight property damage | 161 | 68.20% |
|  | Severe property damage | 75 | 31.80% |
| Physical injury | Yes | 11 | 4.70% |
|  | No | 225 | 95.30% |
| Witness any injuries or deaths | No | 193 | 81.80% |
|  | Yes | 43 | 18.20% |

Note：*n*=237。
