# Supplementary material for: Prevalence and correlates of post-traumatic stress disorder and its symptomatology in tornado-affected rural residents
Source: Front Psychiatry. 2022 Aug 8;13:946450. doi: 10.3389/fpsyt.2022.946450 (PMC9394182; doi:10.3389/fpsyt.2022.946450)
Supplement: Supplementary file 1 [file Data_Sheet_1.ZIP › Date Sheet 1/table 2.docx]

**Table 2.** Results of the Willingness to ask for psychological assistance.

|  |  | **Willingness to ask for psychological assistance** | |  |
| --- | --- | --- | --- | --- |
|  |  | No | Yes | Total |
| **diagnosed with PTSD** | Negative | 198 | 6 | 204 |
|  | Positive | 22 | 10 | 32 |
| Total |  | 220 | 16 | 236 |
