# Supplementary material for: Prevalence and correlates of post-traumatic stress disorder and its symptomatology in tornado-affected rural residents
Source: Front Psychiatry. 2022 Aug 8;13:946450. doi: 10.3389/fpsyt.2022.946450 (PMC9394182; doi:10.3389/fpsyt.2022.946450)
Supplement: Supplementary file 1 [file Data_Sheet_1.ZIP › Date Sheet 1/table 3.docx]

**Table 3.** Results of the chi-square test on collected factors in rural residents who survived the tornado disaster.

| **Factors** |  | | ***n*** | **Number of persons diagnosed with PTSD (%)** | ***X^2^*** | ***P*** |
| --- | --- | --- | --- | --- | --- | --- |
| **Demographic** |  | |  |  |  |  |
| Gender | Male | | 129 | 9（6.98%） | 10.52 | 0.001 |
|  | Female | | 107 | 23（21.50%） |  |  |
| **Exposure to the tornado disaster** |  | |  |  |  |  |
| Property damage | Slight property damage | | 161 | 11（6.83%） | 19.56 | <0.001 |
|  | Severe property damage | | 75 | 21（28.00%） |  |  |
| Physical injury | Yes | | 11 | 6（54.55%） | 16.54 | <0.001 |
|  | No | | 225 | 26（11.56%） |  |  |
| Disaster degree | Living in a mildly affected area | | 141 | 5(3.55%） | 29.96 | <0.001 |
|  | Living in a severely affected area | | 95 | 27（28.42%） |  |  |
| Witness any injuries or deaths | No | 193 | | 22（11.40%） | 4.22 | 0.040 |
|  | Yes | | 43 | 10（23.26%） |  |  |
