# Supplementary material for: Prevalence and correlates of post-traumatic stress disorder and its symptomatology in tornado-affected rural residents
Source: Front Psychiatry. 2022 Aug 8;13:946450. doi: 10.3389/fpsyt.2022.946450 (PMC9394182; doi:10.3389/fpsyt.2022.946450)
Supplement: Supplementary file 1 [file Data_Sheet_1.ZIP › Date Sheet 1/table 4.docx]

**Table 4.** Results of binary logistic regression on factors associated with PTSD in rural residents who survived the tornado disaster.

| **Factors** |  | **OR（95%）** | ***P*** |
| --- | --- | --- | --- |
| **Demographic** |  |  |  |
| Gender | Male |  |  |
|  | Female | 2.82（1.13，7.01） | 0.026 |
| **Exposure to the tornado disaster** |  |  |  |
| Disaster degree | Living in a mildly affected area |  |  |
|  | Living in a severely affected area | 4.51（1.46,13.92） | 0.009 |
| Property damage | Slight property damage |  |  |
|  | Severe property damage | 3.72（1.38,10.05） | 0.010 |
