# Supplementary material for: Prevalence and correlates of post-traumatic stress disorder and its symptomatology in tornado-affected rural residents
Source: Front Psychiatry. 2022 Aug 8;13:946450. doi: 10.3389/fpsyt.2022.946450 (PMC9394182; doi:10.3389/fpsyt.2022.946450)
Supplement: Supplementary file 1 [file Data_Sheet_1.ZIP › Date Sheet 1/table 5.docx]

**Table 5.** PTSD symptoms of rural residents who survived the tornado disaster.

| **Symptom** | ***n*（often or very often）** | **%** |
| --- | --- | --- |
| **Intrusion** |  |  |
| Did the event cause upset thoughts or images that came to your mind although you didn’t want them to? | 18 | 56.20% |
| Did you have nightmares about the event? | 13 | 40.70% |
| Has it ever happened to you that you suddenly felt like you were living through the event again? | 10 | 31.30% |
| Did you feel emotionally upset when you were reminded of the event (feeling helpless, angry, sad, guilty)? | 24 | 75.00% |
| Have you ever had physical reactions when you were reminded of the event (e.g., uneasiness, chills, or fast heartbeat)? | 16 | 50.10% |
| **Avoidance** |  |  |
| Have you tried not to think about the event, not to talk about it, or to suppress feelings about it? | 15 | 46.90% |
| Did you try to avoid situations that remind you of the event (e.g., activities, people, or places)? | 18 | 56.30% |
| Were you unable to remember an important part of the event? | 9 | 28.10% |
| Did you lose interest in activities that had been important to you before the event took place (e.g., hobbies, sports)? |  |  |
| Did you feel alienated or isolated from people in your environment? | 7 | 21.90% |
| Did you feel emotionally numb (e.g., being unable to cry or unable to have positive feelings)? | 10 | 31.20% |
| Did you feel like your plans for the future and hopes would not come true (e.g., to start a family, less luck in life or in business than the others)? | 12 | 37.50% |
| **Hyperarousal** |  |  |
| Did you have trouble falling or staying asleep? | 21 | 65.60% |
| Did you have fits of rage or were you often nervous? | 14 | 43.70% |
| Did you have trouble concentrating (e.g., forgetting what you just wanted to do or forgetting what you just read or what you saw on television)? | 13 | 40.60% |
| Were you overly alert (e.g., checking to see who is around you, having a phone close-by to call for help if necessary)? | 24 | 75.10% |
| Were you easily startled or highly nervous (e.g., by loud noises)? | 19 | 59.40% |
